# Comparison of the measurement properties of the EQ-5D-5L and SF-6Dv2 among overweight and obesity populations in China

**DOI:** 10.1186/s12955-023-02202-1

**Published:** 2023-10-31

**Authors:** Shitong Xie, Meixuan Li, Dingyao Wang, Tianqi Hong, Weihua Guo, Jing Wu

**Affiliations:** 1 School of Pharmaceutical Science and Technology, Tianjin University, Tianjin, China; 2 Center for Social Science Survey and Data, Tianjin University, Tianjin, China; 3 Evidence-based Medicine Center, School of Basic Medical Sciences, Lanzhou University, Lanzhou, Gansu China; 4 School of Biomedical Engineering, McMaster University, Hamilton, ON Canada

**Keywords:** Health-related quality of life, Health state utility value, EQ-5D-5L, SF-6Dv2, Measurement properties, Overweight, Obesity

## Abstract

**Objective:**

To evaluate and compare the measurement properties of the EQ-5D-5L and SF-6Dv2 among Chinese overweight and obesity populations.

**Methods:**

A representative sample of Chinese overweight and obesity populations was recruited stratified by age, gender, body mass index (BMI), and area of residence. Social-demographic characteristics and self-reported EQ-5D-5L and SF-6Dv2 responses were collected through the online survey. The agreement was assessed using intraclass correlation coefficients (ICC). Convergent validity and known-group validity were examined using Spearman’s rank correlation and effect sizes, respectively. The test-retest reliability was assessed using among a subgroup of the total sample. Sensitivity was compared using relative efficiency and receiver operating characteristic.

**Results:**

A total of 1000 respondents (52.0% male, mean age 51.7 years, 67.7% overweight, 32.3% obesity) were included in this study. A higher ceiling effect was observed in EQ-5D-5L than in SF-6Dv2 (30.6% vs. 2.1%). The mean (SD) utility was 0.851 (0.195) for EQ-5D-5L and 0.734 (0.164) for SF-6Dv2, with the ICC of the total sample was 0.639 (p < 0.001). The Spearman’s rank correlation (range: 0.186–0.739) indicated an acceptable convergent validity between the dimensions of EQ-5D-5L and SF-6Dv2. The EQ-5D-5L showed basically equivalent discriminative capacities with the SF-6Dv2 (ES: 0.517–1.885 vs. 0.383–2.329). The ICC between the two tests were 0.939 for EQ-5D-5L and 0.972 for SF-6Dv2 among the subgroup (N = 150). The SF-6Dv2 had 3.7–170.1% higher efficiency than the EQ-5D-5L at detecting differences in self-reported health status, while the EQ-5D-5L was found to be 16.4% more efficient at distinguishing between respondents with diabetes and non-diabetes.

**Conclusions:**

Both the EQ-5D-5L and SF-6Dv2 showed comparable reliability, validity, and sensitivity when used in Chinese overweight and obesity populations. The two measures may not be interchangeable given the systematic difference in utility values between the EQ-5D-5L and SF-6Dv2. More research is needed to compare the responsiveness.

## Introduction

Overweight and obesity have become a major global public health issue. Rates of overweight and obesity have increased rapidly in the past four decades [1]. According to WHO statistic in 2016, more than 1.9 billion people aged ≥ 18 years are overweight around the world, of these over 650 million are obese [2]. According to the Report on Chinese Residents’ Chronic Diseases and Nutrition 2020, more than half of the Chinese adults had either overweight or obesity [1]. Overweight and obesity contributed to 11.1% of deaths associated with noncommunicable diseases (NCDs) in 2019 worldwide, with a rapid increase from 5.7% in 1990 [1]. These conditions also incurred substantial national health expenditure for the management of NCDs, and has also been shown to negatively impact health-related quality of life (HRQoL) [3].

HRQoL has been extensively used worldwide as a multidimensional concept that could be used to assess an individual’s health status based on physical, mental, and social functioning [4]. The European Medicines Agency [5] and the US Food and Drug Administration [6] have emphasized the importance of measuring HRQoL, which is considered an important piece of evidence to inform drug coverage or reimbursement decisions in many countries [7, 8]. Health-related quality of life (HRQoL) measures can be categorized as either non–preference-based or preference-based measures [9, 10]. Preference-based HRQoL measures can be used to elicit health state utility values (HSUVs) that take into account the preference on different health states by the general population and lie on a 0 to 1 (death to full health) quality-adjusted life-years (QALYs) scale [11].

Currently, the EQ-5D and the Short Form Six-Dimension (SF-6D) are the two most widely used generic preference-based measures (GPBMs) [12] and are recommended as the standard measures in the application of health technology assessment in many countries [13–15]. The measurement properties of the EQ-5D and SF-6D have been evaluated in the general population as well as patients with various types of diseases [16–32]. These studies concluded that the EQ-5D and SF-6D were generally reliable, valid, and sensitive to measuring HSUVs in various disease populations. However, it should be noted that most of the above studies has not compared the test-retest reliability, an important psychometric property of the GPBMs. More importantly, evidence evaluating the measurement properties of the GPBMs in the overweight and obesity populations is still lacking worldwide. To the best of our knowledge, no studies have evaluated and compared the measurement properties of the EQ-5D-5L and SF-6Dv2 among overweight and obesity populations.

This study aimed to assess and compare the measurement properties of the EQ-5D-5L and SF-6Dv2 in Chinese overweight and obesity populations.

## Methods

### Data source

The data used for this analysis were obtained from a nationwide online survey (from Jan to Feb 2022) investigating the health status of people living with overweight or obesity in China. Recruitment of the respondents was conducted through a professional online panel company. Inclusion criteria were that respondents (1) were 18 years or older; (2) overweight (24 ≤ BMI<28) or obese (BMI ≥ 28) according to criteria of overweight and obesity for the Chinese populations [33]; (3) were literate and able to read text from a computer or mobile screen, and had no disease limiting cognitive function such as dementia; and (4) gave informed consent. A quota sampling method was also used to recruit a representative sample of the overweight and obese populations in terms of BMI, age, gender, area of residence (North, Northeast, East, Central, South, Southwest, Northwest) [34].

All eligible respondents (target N = 1,000) were invited to complete a self-reported online survey through computer or mobile phone. Information on social-demographic including ethnicity, education level, marital status, employment status, personal monthly income, health insurance coverage; health-related questions including a 5-level categorized self-reported health status (very good, good, fair, bad, very bad), presence of chronic diseases, smoking and alcohol consumption status, fruit and vegetable intake, high-fat and high-sugar food intake and weekly exercise time; and the EQ‑5D‑5L and SF-6Dv2 self-reported answers were collected. The order of the EQ‑5D‑5L and SF-6Dv2 was randomized.

A subset of respondents (target N = 150) was recruited to assess the test-retest reliability of both instruments. After the first survey (test), the interviewers randomly asked for the respondents’ consent to be online interviewed again (retest) and collected the contact information. The interval between the test and retest was set as two weeks [35, 36]. In the retest interview, respondents completed the same process as in the first interview. During the retest interview, the respondent was asked the question “Have there been any changes in your health status compared with the last interview?” and rated on a 5-level Likert scale (“no change”, “slightly change”, “some change”, “much change”, or “extremely change”). The respondents who reported “no change”, “slightly change” were regarded to have relatively stable health over the two tests and included in the data analysis [37, 38].

### Measures

The EQ-5D-5L descriptive system measures health along five dimensions including mobility, self-care, usual activities, pain/discomfort and anxiety/depression. Each dimension is assessed by a single question on a five-point ordinal scale from no problem to extreme problems [39]. The other part of EQ-5D-5L is a visual analog scale (hereafter EQ VAS), which is a vertical line with endpoints of ‘‘worst imaginable health’’ at 0 and ‘‘best imaginable health’’ at 100. The EQ-5D-5L defines 3,125 unique health states, with 11111 being the best health state (full health), and 55555 the worst health state. The time trade-off (TTO) approach was used to develop the Chinese EQ-5D-5L utility value set, with utility values ranging from − 0.391 (55555) to 1 (11111) [40].

The SF-6Dv2 is a revised version of the SF-6Dv1 that is derived from 10 items of the SF-36v2. The SF-6Dv2 health state classification system measured on six dimensions, including physical functioning, role limitation, social functioning, pain, mental health, and vitality. The pain dimension has six response levels, while all others have five levels. Overall the SF-6Dv2 descriptive system can define 18,750 (= 5*5*5*6*5*5) unique health states [41]. The Chinese SF-6Dv2 value set was developed using the TTO approach, with the utility values ranged from − 0.277 (555655) to 1 (111111) [42].

Both validated Chinese versions of EQ-5D-5L and SF-6Dv2 were used in this study [32, 37].

### Statistical analysis

#### Descriptive statistics

Descriptive statistics were used to describe the characteristics of respondents, and utility values of the two instruments. The differences between test and retest respondents’ characteristics were tested using the ANOVA for continuous variables and chi-squared test for categorical variables and presented within tables. The distribution of response levels on each dimension of the EQ-5D-5L and SF-6Dv2 was reported using histograms.

#### Agreement

The intraclass correlation coefficient (ICC) was used to investigate the agreement between EQ-5D-5L and SF-6Dv2. The ICC was computed with the two-way mixed-effects model based on absolute agreement [43]. An ICC above 0.7 suggests an acceptable agreement [44]. Besides, because the utility value distributions were highly skewed, the Wilcoxon signed-rank test was used to compare the utility values of the EQ-5D-5L and SF-6Dv2 [45].

#### Measurement properties of the EQ-5D-5L and SF-6Dv2

We focused on the aspects of ceiling and floor effects, convergent validity, known-group validity, test-retest reliability, and sensitivity that are important for assessing the performance of measurement properties of the preference-based measures.

*Ceiling and floor effects*. We evaluated ceiling and floor effects for the EQ-5D-5L and SF-6Dv2 by examining the percentage of respondents who reported the best and worst possible health states, respectively. Ceiling or floor effects were considered to be present if more than 15% of the respondents achieved either extreme end of the scale [46].

*Convergent validity*. Convergent validity was assessed by calculating Spearman’s rank coefficient (r) between the EQ-5D-5L and SF-6Dv2 dimensions. An absolute coefficient value greater than 0.5 stands for a strong correlation, values between 0.35 and 0.49 for moderate, values between 0.2 and 0.34 for weak, and values smaller than 0.2 for poor correlation [17, 32, 47].

*Known-group validity*. Known-group validity was used to assess the extent to which an outcome measure of interest helps distinguish between sub-groups that are theoretically expected to differ [20, 32]. Based on the published literature [32, 45, 48], it was hypothesized that the obese respondents, as well as respondents with poorer self-reported health status and more chronic diseases, had lower utility values. One-way analysis of variance (ANOVA) and Scheffe post hoc test to analyze possible differences in utility values of the EQ-5D-5L and SF-6Dv2 across different sub-groups. Besides, effect sizes (ES) were also used to define the discriminative capacity of the EQ-5D-5L and SF-6Dv2, which were calculated as the difference between the mean utility of two sub-groups divided by the pooled standard deviation. For polytomous variables, the ES between the extreme sub-groups (e.g., the ES between the sub-group with no chronic disease and the sub-group with ≥ 4 chronic diseases) were calculated [32, 48]. Generally, an ES value of 0.20 is defined as small, 0.50 as medium, and 0.80 as large.

*Test-retest reliability*. The test-retest reliability of the EQ-5D-5L and SF-6Dv2 was evaluated using the test and retest data by the intra-class correlation coefficient (ICC), which was computed with the two-way mixed-effects model based on absolute agreement. ICC value above 0.7 was considered as satisfactory reliability [49].

*Sensitivity*. The relative efficiency (RE) statistic was used to assess the sensitivity of the EQ-5D-5L and SF-6Dv2 for detecting differences in both external and self-reported health indicators. RE was calculated via the ratio of the square of t-statistics from the t-tests of the comparator measure (SF-6Dv2) over that of the reference measure (EQ-5D-5L) [50, 51]. A RE value of 1.0 indicates that the SF-6Dv2 has the same efficiency as EQ-5D-5L at detecting differences. A value higher than 1 indicates that the SF-6Dv2 is more sensitive than the EQ-5D-5L, while a value lower than 1 means the opposite [52]. The sensitivity of these two measures was also assessed using the receiver operating characteristic (ROC) curve [53]. To compare the discriminative power of the EQ-5D-5L and SF-6Dv2, the area under the ROC curve (AUC) was calculated [54]. The one with the larger AUC is thought to be more sensitive or effective at detecting differences, and measures with excellent discriminative ability would have an AUC score of 1.0, whereas measures with no discriminative capacity would have an AUC score of 0.5 [52]. The presence of representative chronic diseases, including hyperlipidemia, hypertension and diabetes, among overweight and obesity populations was used as external health indicators in the current study [55, 56]. The respondents’ self-reported health status was divided into three categories: (1) excellent versus good, fair, or bad, (2) excellent or good versus fair or bad, and (3) excellent, good, or fair versus bad.

STATA 15.0 was used for the statistical analyses (StataCorp LLC, College Station, TX, USA). All statistical tests reported were two-sided with a significance level of 0.05.

## Results

### Patient characteristics

A total of 9,085 potential respondents were reached out in the first round of survey (according to geographical region, gender and age quota), of which 8,259 respondents agreed to participate (the response rate was 90.9%). Among them, 7,088 respondents withdrew passively because they did not meet the BMI quota requirements (not overweight/obese [5,911] or the quota was full [1,177]), and 171 respondents voluntarily withdrew from the process of filling in the questionnaire. Finally, a total of 1,000 respondents with valid data were included in this study.

As shown in Tables 1 and 52.0% (N = 520) of respondents were male, and the mean (SD) age was 51.7 (15.3) years, with a range from 18 to 80 years, and 29.3% (N = 293) of respondents were more than 65 years old. The mean (SD) BMI of respondents was 27.4 (2.8), of which 67.7% (N = 677) were overweight with 24 ≤ BMI < 28, and 32.3% (N = 323) were obesity with BMI ≥ 28. 32.7% (N = 327), 29.2% (N = 292), and 8.9% (N = 89) of respondents had hyperlipidemia, hypertension, and diabetes, respectively.

**Table 1:** Characteristics of respondents

Characteristics	Total sample (N = 1,000) N (%)	Test-retest sample (N = 150) N (%)	P value*
**Gender**			0.215
Male	520 (52.0%)	85 (56.7%)	
Female	480 (48.0%)	65 (43.3%)	
**Age (mean [SD])**	51.7 (15.3)	50.6 (15.1)	0.789
**Age group (years)**			0.306
18–34	174 (17.4%)	28 (18.7%)	
35–44	162 (16.2%)	26 (17.3%)	
45–54	192 (19.2%)	27 (18.0%)	
55–64	179 (17.9%)	34 (22.7%)	
≥ 65	293 (29.3%)	35 (23.3%)	
**Residence (Geographical division)**			0.710
North	184 (18.4%)	25 (16.7%)	
Northeast	173 (17.3%)	22 (14.7%)	
East	134 (13.4%)	20 (13.3%)	
Central	136 (13.6%)	22 (14.7%)	
South	96 (9.6%)	20 (13.3%)	
Southwest	131 (13.1%)	20 (13.3%)	
Northwest	146 (14.6%)	21 (14.0%)	
**BMI (mean [SD])**	27.4 (2.8)	27.2 (2.7)	0.814
**BMI**			0.158
24 ≤ BMI<28	677 (67.7%)	109 (72.7%)	
BMI ≥ 28	323 (32.3%)	41 (27.3%)	
**Residence**			0.602
Urban area	832 (83.2%)	127 (84.7%)	
Rural area	168 (16.8%)	23 (15.3%)	
**Ethnic group**			0.745
Han	977 (97.7%)	146 (97.3%)	
Minority	23 (2.3%)	4 (2.7%)	
**Education**			0.207
Primary or below	196 (19.6%)	22 (14.7%)	
Junior high school	312 (31.2%)	43 (28.7%)	
Senior high school	338 (33.8%)	58 (38.7%)	
College or above	154 (15.4%)	27 (18.0%)	
**Marital status**			**0.037**
Unmarried	81 (8.1%)	20 (13.3%)	
Married	890 (89.0%)	126 (84.0%)	
Divorced	12 (1.2%)	3 (2.0%)	
Widowed	17 (1.7%)	1 (0.7%)	
**Employment status**			0.988
Employed	683 (68.3%)	103 (68.7%)	
Retired	284 (28.4%)	42 (28.0%)	
Student	11 (1.1%)	2 (1.3%)	
Unemployed	22 (2.2%)	3 (2.0%)	
**Personal monthly income**			0.672
<2000 RMB	70 (7.0%)	11 (7.3%)	
2000–5000 RMB	386 (38.6%)	52 (34.7%)	
5000–10,000 RMB	444 (44.4%)	69 (46.0%)	
>10,000 RMB	100 (10.0%)	18 (12.0%)	
**Basic medical insurance**			0.750
Urban employee	811 (81.1%)	125 (83.3%)	
Urban and rural resident	174 (17.4%)	23 (15.3%)	
No	15 (1.5%)	2 (1.3%)	
**Commercial insurance**			0.235
Yes	88 (8.8%)	17 (11.3%)	
No	912 (91.2%)	133 (88.7%)	
**Self-report health status**			0.898
Poor	167 (16.7%)	24 (16.0%)	
General	440 (44.0%)	63 (42.0%)	
Good	314 (31.4%)	51 (34.0%)	
Very good	79 (7.9%)	12 (8.0%)	
**Hypertension**			0.969
Yes	292 (29.2%)	44 (29.3%)	
No	708 (70.8%)	106 (70.7%)	
**Diabetes**			0.608
Yes	89 (8.9%)	15 (10.0%)	
No	911 (91.1%)	135 (90.0%)	
**Hyperlipidemia**			0.183
Yes	327 (32.7%)	42 (28.0%)	
No	673 (67.3%)	108 (72.0%)	
**Number of chronic diseases**			0.276
0	410 (41.0%)	66 (44.0%)	
1	182 (18.2%)	22 (14.7%)	
2	169 (16.9%)	30 (20.0%)	
3	96 (9.6%)	9 (6.0%)	
≥ 4	143 (14.3%)	23 (15.3%)	
**Weight loss therapy**			**0.017**
Yes	231 (23.1%)	46 (30.7%)	
No	769 (76.9%)	104 (69.3%)	
**Smoking status**			0.357
Never smoked	588 (58.8%)	85 (56.7%)	
Used to smoke	239 (23.9%)	33 (22.0%)	
Smoking now	173 (17.3%)	32 (21.3%)	
**Drinking status**			0.188
Never drink	393 (39.3%)	69 (46.0%)	
Used to drink	243 (24.3%)	33 (22.0%)	
Drinking now	364 (36.4%)	48 (32.0%)	
**Exercise duration/week**			0.455
≤ 3.5 h	568 (56.8%)	81 (54.0%)	
3.5-7.5 h	395 (39.5%)	61 (40.7%)	
≥ 7.5 h	37 (3.7%)	8 (5.3%)	
**Fruit and vegetable intake**			0.650
Rarely intake	174 (17.4%)	30 (20.0%)	
Sometimes intake	338 (33.8%)	50 (33.3%)	
Often intake	488 (48.8%)	70 (46.7%)	
**High sugar oil food intake**			0.935
Rarely intake	152 (15.2%)	22 (14.7%)	
Sometimes intake	473 (47.3%)	73 (48.7%)	
Often intake	375 (37.5%)	55 (36.7%)	
**Sleep duration (day)**			0.077
< 7 h	579 (57.9%)	77 (51.3%)	
≥ 7 h	421 (42.1%)	73 (48.7%)	

Note:* difference between subgroups within the same classification; p value significant < 0.05

The difference between scores, characteristics and utility values were tested using the ANOVA for continuous variables and chi-squared test for categorical variables

The distribution of the responses to the EQ-5D-5L and SF-6Dv2 are presented in Fig. 1. For EQ-5D-5L, 30.6% of respondents reported full health, which indicated a significant ceiling effect; while for SF-6Dv2, no ceiling effect was obverted with 2.1% of respondents reported no problems on all dimensions. No respondent reported the worst health state for both measures.

**Fig. 1a:**
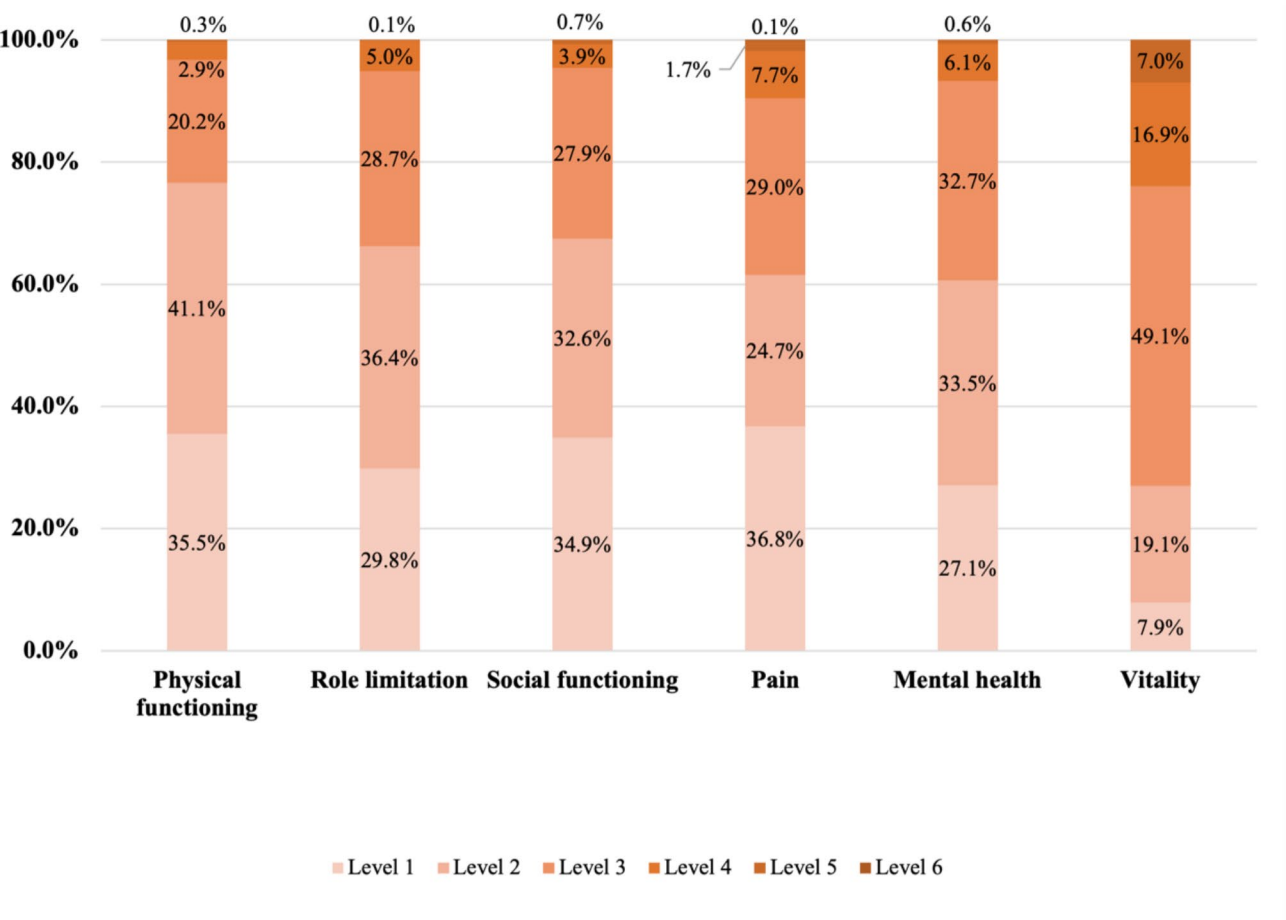
Distribution across levels of the EQ-5D-5L dimensions

**Fig. 1b:**
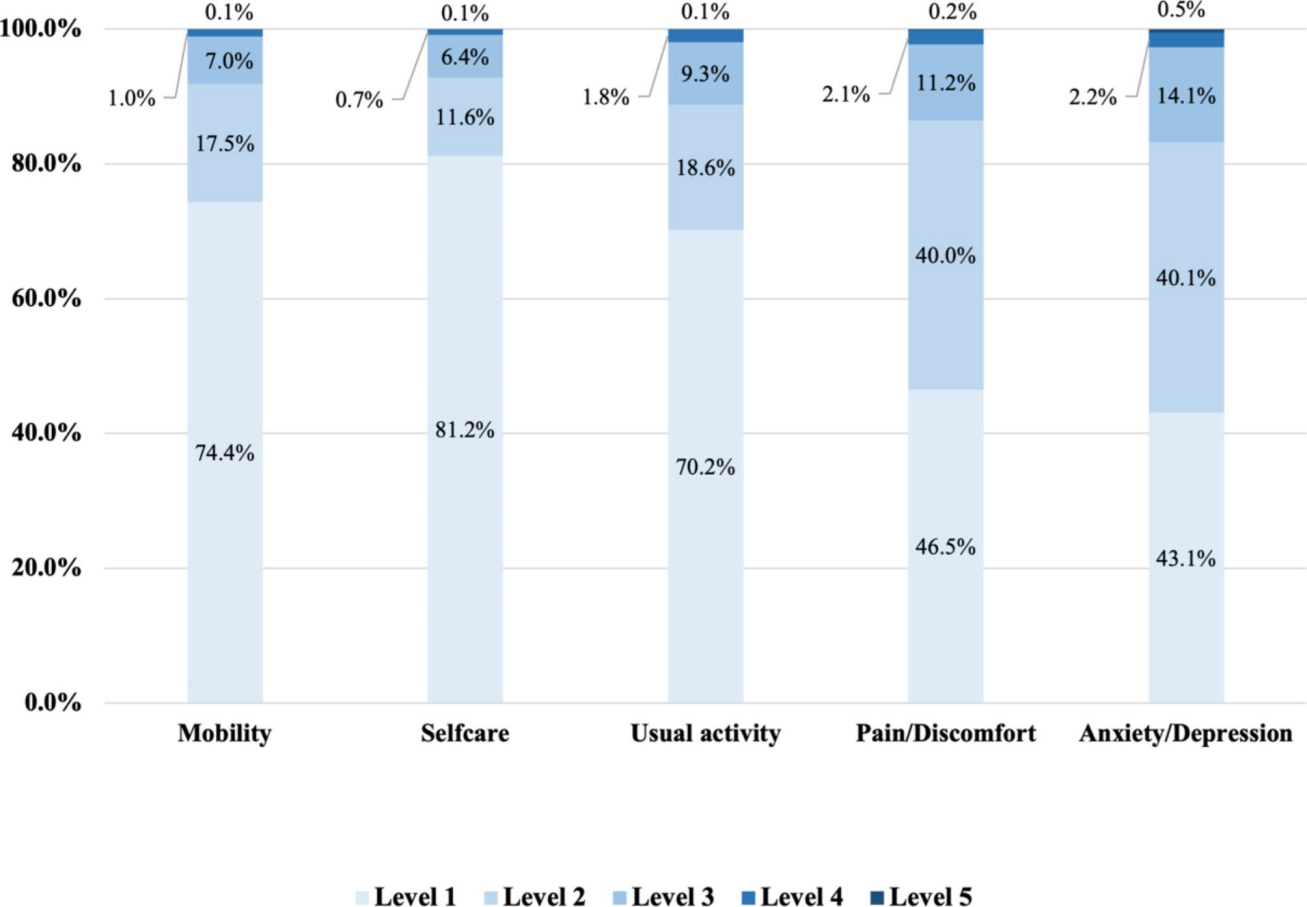
Distribution across levels of the SF-6Dv2 dimensions

The mean (SD) EQ-5D-5L utility value among the total sample was 0.851 (0.195), ranging from − 0.184 to 1, and mean SF-6Dv2 utility was 0.734 (SD = 0.164), ranging from − 0.179 to 1. For the overweight respondents with 24 ≤ BMI < 28, mean EQ-5D-5L utility was 0.880, and mean SF-6Dv2 utility was 0.754; For the obesity respondents with BMI ≥ 28, mean EQ-5D-5L utility was 0.789, and mean SF-6Dv2 utility was 0.694.

### Agreement

The ICC between the EQ-5D-5L and SF-6Dv2 utility values of the total sample was 0.639 (p < 0.001). Besides, the SF-6Dv2 utility values were significantly lower than those of the EQ-5D-5L (p < 0.001).

### Measurement properties of the EQ‑5D‑5L and SF‑6Dv2

*Ceiling and floor effects.* A ceiling effect was found for the EQ-5D-5L, with the proportion of respondents reporting the best health state was 30.6% (N = 306), while no floor effects was observed. No ceiling or floor effects were observed in the SF-6Dv2.

*Convergent validity*. Most of the dimensions of EQ-5D-5L and SF-6Dv2 were positively and associated, with Spearman’s rank correlation coefficient ranging from 0.186 to 0.739 (p < 0.001); As expected, the EQ-5D-5L pain/discomfort dimension was strongly correlated with the SF-6Dv2 pain dimension (r = 0.739), and the EQ-5D-5L anxiety/depression dimension was highly correlated with the SF-6Dv2 mental health dimension (r = 0.686). The correlation between SF-6Dv2 vitality dimension and all dimensions of EQ-5D-5L was weak (Table 2).

**Table 2:** Correlations between EQ-5D-5L and SF-6Dv2 (N = 1,000)

SF-6Dv2	EQ-5D-5L				
	Mobility	Self-care	Usual activity	Pain/Discomfort	Anxiety/Depression
**Physical functioning**	0.469	0.402	0.475	0.497	0.382
**Role limitation**	0.470	0.415	**0.501**	**0.568**	**0.571**
**Social functioning**	0.459	0.395	0.482	**0.520**	**0.544**
**Pain**	0.448	0.381	0.465	**0.739**	**0.502**
**Mental health**	0.387	0.338	0.424	**0.522**	**0.686**
**Vitality**	0.237	0.186	0.251	0.225	0.233

**Note:** r > 0.5 represents a strong correlation

All the p values of the correlations were lower than 0.001

*Known-group validity*. As reported in Table 3, both the EQ-5D-5L and SF-6Dv2 utility values were significantly different (p < 0.001) across groups defined by BMI, health status, and number of chronic diseases, with ES ranging from 0.517 to 1.885 for the EQ-5D-5L, and 0.383–2.329 for the SF-6Dv2. The hypotheses for known-group validity were fulfilled in all tested groups, that is, the obese respondents, as well as respondents with poorer self-reported health status and more chronic diseases, had lower utility values.

**Table 3:** Discriminative capacity and univariate analyses for EQ-5D-5L and SF-6Dv2 utility among different sub-groups (N = 1,000)

	EQ-5D-5L				SF-6Dv2			
	Mean (SD)	p value	Scheffe post hoc test	Effect size ^a^ (95% CI)	Mean (SD)	p value	Scheffe post hoc test	Effect size ^a^ (95% CI)
**BMI group**		< 0.001		0.517 (0.327, 0.706)		< 0.001		0.383 (0.194, 0.571)
I: 24 ≤ BMI < 26 (N = 406)	0.882 (0.165)		I > III***, I > IV***, II > III***, II > IV***		0.756 (0.153)		I > III**, I > IV**, II > III**, II > IV**	
II: 26 ≤ BMI < 28 (N = 271)	0.876 (0.160)				0.749 (0.137)			
III: 28 ≤ BMI < 30 (N = 151)	0.784 (0.248)				0.693 (0.191)			
IV: BMI ≥ 30 (N = 172)	0.794 (0.230)				0.694 (0.187)			
**Health status**		< 0.001		1.717 (1.407, 2.023)		< 0.001		2.329 (1.989, 2.625)
I: Excellent (n = 79)	0.986 (0.036)		I > III***, I > IV***, II > III***, II > IV***, III > IV***		0.915 (0.101)		I > II***, I > III***, I > IV***, II > III***, II > IV***, III > IV***	
II: Good (n = 314)	0.944 (0.086)				0.830 (0.103)			
III: Fair (n = 440)	0.846 (0.166)				0.707 (0.108)			
IV: Bad (n = 167)	0.622 (0.256)				0.540 (0.183)			
**Number of chronic diseases**		< 0.001		1.885 (1.664, 2.105)		< 0.001		2.076 (1.850, 2.303)
I: 0 (N = 410)	0.940 (0.112)		I > II**, I > III***, I > IV***, I > V***, II > III**, II > IV***, II > V***, III > V***, IV > V***		0.835 (0.116)		I > II***, I > III***, I > IV***, I > V***, II > III**, II > IV***, II > V***, III > V***, IV > V***	
II: 1 (N = 182)	0.892 (0.137)				0.743 (0.119)			
III: 2 (N = 169)	0.819 (0.147)				0.683 (0.118)			
IV: 3 (N = 96)	0.773 (0.202)				0.656 (0.114)			
V: ≥4 (N = 143)	0.630 (0.262)				0.548 (0.189)			

**Note:** One-way analyses of variance and Scheffe post hoc tests were performed to compare the EQ-5D-5L and SF-6Dv2 utility among different sub-groups

The effect size was calculated as the difference between the mean scores of two sub-groups divided by the pooled standard deviation. An effect size of 0.8 is defined as large, 0.5 to 0.79 as moderate, and 0.2 to 0.49 as small. **Abbr**: BMI: Body Mass Index, equals weight(kg) divided by height(m) squared. BMI groups were defined according to the guideline published by the Cooperative Meta-analysis Group of China Obesity Task Force in 2002

*Test-retest reliability*. Among 227 respondents who were invited to attend the retest interview, 220 respondents accepted the invitation with a response rate of 96.9%. 150 respondents who reported “no change” and “slightly change” in their health status compared with the last interview provided valid test–retest data. As shown in Table 1, the majority of the respondents were male (56.7%), mean (SD) age of 50.6 (15.1) years. Except for marital status, no significant difference was obverted in basic characteristics between the 150 respondents and total sample. Both instruments showed good test-retest reliability. For the EQ-5D-5L, the overall ICC was 0.939 (95% CI 0.917, 0.955), where for overweight was 0.933 (95% CI 0.903, 0.954), and obese was 0.941 (95% CI 0.890, 0.969). For the SF-6Dv2, the overall ICC was 0.972 (95% CI 0.962, 0.980), where overweight was 0.980 (95% CI 0.971, 0.986), and obese was 0.954 (95% CI 0.916, 0.975).

*Sensitivity*. As shown in Table 4, the SF-6Dv2 had 3.7-170.1% higher efficiency at revealing differences between self-reported health status groups dichotomized by “excellent”, “good” or “bad”. The SF-6Dv2 was also found to be 26.1% and 44.7% more efficient than the EQ-5D-5L at detecting differences in external health indicator hyperlipidemia and hypertension groups, respectively. However, when the groups were dichotomized by “diabetes” and “non-diabetes”, the EQ-5D-5L was found to be 16.6% more efficient at detecting differences in external health indicator groups (Table 5). The AUC values of both SF-6Dv2 and EQ- 5D-5L were above 0.5 with statistically significant differences (p < 0.001) (Tables 4 and 5). The SF-6Dv2 generated higher AUC scores than the EQ-5D-5L, indicating a possible sensitivity superiority.

**Table 4:** Sensitivity of EQ-5D-5L and SF-6Dv2 to detect differences in different self-reported health status groups (N = 1,000)

Measurement	Categorisation of different self-reported health status groups	N	Utility value (Mean [SD])	t-test		RE^b^	ROC curve	
				t-statistic	p-value		AUC	95% CI
EQ-5D-5L	excellent	79	0.986 (0.036)	-6.546	< 0.001	1.000	0.815*	(0.784, 0.846)
	good, fair or bad	921	0.839 (0.199)					
SF-6Dv2	excellent	79	0.915 (0.101)	-10.758	< 0.001	2.701	0.879*	(0.841, 0.917)
	good, fair or bad	921	0.719 (0.159)					
EQ-5D-5L	excellent or good	393	0.952 (0.080)	-14.614	< 0.001	1.000	0.801*	(0.774, 0.827)
	fair or bad	607	0.785 (0.218)					
SF-6Dv2	excellent or good	393	0.847 (0.108)	-21.046	< 0.001	2.074	0.862*	(0.839, 0.884)
	fair or bad	607	0.661 (0.152)					
EQ-5D-5L	excellent, good or fair	833	0.896 (0.142)	19.478	< 0.001	1.000	0.862*	(0.831, 0.893)
	bad	167	0.622 (0.256)					
SF-6Dv2	excellent, good or fair	833	0.773 (0.128)	19.834	< 0.001	1.037	0.881*	(0.853, 0.909)
	bad	167	0.540 (0.183)					

**Note:** *p < 0.001. For the ROC curve, p < 0.001 indicates that AUC is statistically significantly greater than 0.5 and that measure has discriminatory power

a RE of SF-6Dv2 is presented, and reference is EQ-5D-5L, of which RE is 1.000

**Abbr:** AUC Area under the ROC curve, 95% CI 95% confidence interval, RE Relative efficiency, ROC Receiver operating characteristic, SD Standard deviation

**Table 5:** Sensitivity of EQ-5D-5L and SF-6Dv2 to detect differences in different chronic diseases groups (N = 1,000)

Measurement	Categorisation of different chronic diseases groups	N	Utility value (Mean[SD])	t-test		Relative effiency^a^	ROC curve	
				t-statistic	p-value		AUC	95% CI
EQ-5D-5L	Hypertension	292	0.732 (0.242)	13.346	< 0.001	1.000	0.754^*^	(0.721, 0.786)
	Non-hypertension	708	0.899 (0.147)					
SF-6Dv2	Hypertension	292	0.619 (0.169)	16.052	< 0.001	1.447	0.793^*^	(0.763, 0.822)
	Non-hypertension	708	0.782 (0.136)					
EQ-5D-5L	Diabetes	89	0.606 (0.293)	13.469	< 0.001	1.000	0.803^*^	(0.751, 0.854)
	Non-diabetes	911	0.874 (0.165)					
SF-6Dv2	Diabetes	89	0.544 (0.215)	12.298	< 0.001	0.834	0.801^*^	(0.754, 0.849)
	Non-diabetes	911	0.752 (0.146)					
EQ-5D-5L	Hyperlipidemia	327	0.741 (0.236)	13.411	< 0.001	1.000	0.762^*^	(0.732, 0.793)
	Non-hyperlipidemia	673	0.904 (0.145)					
SF-6Dv2	Hyperlipidemia	327	0.633 (0.169)	15.057	< 0.001	1.261	0.775^*^	(0.745, 0.805)
	Non-hyperlipidemia	673	0.783 (0.137)					

**Note:** *p < 0.001. For the ROC curve, p < 0.001 indicates that AUC is statistically significantly greater than 0.5 and that measure has discriminatory power

a RE of SF-6Dv2 is presented, and reference is EQ-5D-5L, of which RE is 1.000

**Abbr:** AUC Area under the ROC curve, 95% CI 95% confidence interval, RE Relative efficiency, ROC Receiver operating characteristic, SD Standard deviation

## Discussion

To the best of our knowledge, this study provided the first evidence of comparing the measurement properties between the EQ-5D-5L and SF-6Dv2 in a large sample of the Chinese overweight and obesity populations. This study could facilitate medical or public health professionals and regulators to understand and select the appropriate measure to make decisions in overweight and obesity clinical interventions and policies.

The EQ-5D-5L showed an higher ceiling effect than the SF-6Dv2 in this study (30.6% vs. 2.1%), which is consistent with previous studies where the EQ-5D-5L and SF-6D were compared in both general and disease populations [18, 32, 57, 58]. This can be partly explained by the difference in the recall period, as the SF-6D frames its questions in terms of health “over the last 4 weeks”, while “today” is used in EQ-5D. A longer recall period may provide more scopes for respondents to include small impaired issues affecting their HRQoL that might not be detected during a relatively short period [59]. Another justification might be a strong relationship with the dimensions and items measured [32, 37].

Both the EQ-5D-5L and SF-6Dv2 were found to have an acceptable reliability and internal consistency. The SF-6Dv2 (ICC = 0.972) performs better than EQ-5D-5L (0.939) in terms of test-retest reliability, implying SF-6Dv2 has ability to produce reproducible results from patients if the instrument is used repeatedly within a short period of time. This finding appears to be consistent with one previous study [60]. Regarding convergent validity, as expected, only the EQ-5D-5L pain/discomfort and anxiety/depression dimensions were strongly correlated with the SF-6Dv2 pain and mental health dimension. The correlation between the SF-6Dv2 vitality dimension and all dimensions of EQ-5D-5L were weak. A possible reason for this could be the fact that the EQ-5D-5L has four out of five items assessing physical health, whereas the SF-6D consists of a balanced number of physical and mental items. Our findings are consistent with previous studies [28, 61], implying that the EQ-5D-5L is appropriate for applying to patients with more physical problems than those with mental or psychological problems.

Known-group validity indicated that both the EQ-5D-5L and SF-6Dv2 were able to discriminate between populations with different levels of self-reported health status and different number of chronic diseases that were expected. These differences tended to be more apparent for the SF-6Dv2 with larger effects sizes (ES = 1.717–1.885 for EQ-5D-5L and 2.076–2.329 for SF-6Dv2). One of the possible reasons is that the SF-6Dv2 has one more dimension, resulting in a larger descriptive system than EQ-5D-5L (18,750 vs. 3,125 health states). This result was consistent with one previous study, which found that the SF-6D in general showed better sensitivity and construct validity than the EQ-5D-5L in seven diseases [62]. Moreover, although the hypotheses for known-group validity were fulfilled in all tested groups, this study found that both instruments were not sensitive enough (ES < 0.8) to differentiate overweight and obesity respondents in different degrees of severity. This may be explained because the GPBMs may be insensitive to measure specific diseases [63]. More evidence is warranted to assess the use of GPBMs among overweight and obesity populations.

RE and ROC analysis showed that the SF-6Dv2 was more efficient to detect differences between self-reported health status groups, while the EQ-5D-5L was found to be more efficient than the SF-6Dv2 at detecting differences in external health indicator groups. The AUC of SF-6Dv2 (0.775–0.881) was always higher than that of EQ-5D-5L (0.754–0.862) in all tested groups. Possible reasons for this may be related to the differences in the recall period, and the number of dimensions between the two instruments. These findings are consistent with previous studies, which were conducted to compare the SF-6Dv1 or SF-6Dv2 with EQ-5D-5L in the general population and patients with some other types of diseases, and concluded that both instruments are sensitive to different groups [30, 32, 64].

Several limitations of this study should be addressed. First, we only focused on adults while did not include adolescents with high prevalence of overweight and obesity, which may have an impact on the representativeness of overweight and obesity in China. Second, online survey was used in this study, which may affect the quality of collected data. While this concern was addressed by monitoring IP addresses and response time of respondents to ensure the authenticity and validity of the collected data. Third, although we conducted the test-retest based on the longitudinal data, the follow-up duration was relative short to evaluate and compare the responsiveness of EQ-5D-5L and SF-6Dv2. Further research is warranted to compare the responsiveness. Besides, in order to reach a satisfied sample size, respondents who reported “no change”, “slightly change” were regarded to have relatively stable health over the two tests and included in the data analysis. This may have an impact on the test-retest reliability analysis.

## Conclusions

Both the EQ-5D-5L and SF-6Dv2 are psychometrically sound instruments with satisfactory validity, reliability, and sensitivity in measuring the HRQoL of Chinese overweight and obesity populations. While these two measures cannot generally be used interchangeably given the ICC value between the SF-6Dv2 and EQ-5D-5L is moderate and the utility values obtained from the two measures are systematically different.

## Data Availability

Not applicable.
